# The Nutraceutic Silybin Counteracts Excess Lipid Accumulation and Ongoing Oxidative Stress in an *In Vitro* Model of Non-Alcoholic Fatty Liver Disease Progression

**DOI:** 10.3389/fnut.2017.00042

**Published:** 2017-09-19

**Authors:** Giulia Vecchione, Elena Grasselli, Federica Cioffi, Francesca Baldini, Paulo J. Oliveira, Vilma A. Sardão, Katia Cortese, Antonia Lanni, Adriana Voci, Piero Portincasa, Laura Vergani

**Affiliations:** ^1^DISTAV, Department of Earth, Environment and Life Sciences, University of Genova, Genoa, Italy; ^2^Department of Science and Technology, University of Sannio, Benevento, Italy; ^3^Center for Neuroscience and Cellular Biology (CNC), University of Coimbra, Coimbra, Portugal; ^4^Department of Experimental Medicine (DIMES), University of Genova, Genoa, Italy; ^5^Department of Biomedical Sciences and Human Oncology, University of Bari Medical School, Bari, Italy

**Keywords:** non-alcoholic fatty liver disease, non-alcoholic steatohepatitis, FaO hepatoma cells, lipid metabolism, oxidative stress, silybin

## Abstract

Non-alcoholic fatty liver disease (NAFLD) is a major cause of liver-related morbidity and mortality. Oxidative stress and release of pro-inflammatory cytokines, such as tumor necrosis factor α (TNFα), are major consequences of hepatic lipid overload, which can contribute to progression of NAFLD to non-alcoholic steatohepatitis (NASH). Also, mitochondria are involved in the NAFLD pathogenesis for their role in hepatic lipid metabolism. Definitive treatments for NAFLD/NASH are lacking so far. Silybin, the extract of the milk thistle seeds, has previously shown beneficial effects in NAFLD. Sequential exposure of hepatocytes to high concentrations of fatty acids (FAs) and TNFα resulted in fat overload and oxidative stress, which mimic *in vitro* the progression of NAFLD from simple steatosis (SS) to steatohepatitis (SH). The exposure to 50 µM silybin for 24 h reduced fat accumulation in the model of NAFLD progression. The *in vitro* progression of NAFLD from SS to SH resulted in reduced hepatocyte viability, increased apoptosis and oxidative stress, reduction in lipid droplet size, and up-regulation of IκB kinase β-interacting protein and adipose triglyceride lipase expressions. The direct action of silybin on SS or SH cells and the underlying mechanisms were assessed. Beneficial action of silybin was sustained by changes in expression/activity of peroxisome proliferator-activated receptors and enzymes for FA oxidation. Moreover, silybin counteracted the FA-induced mitochondrial damage by acting on complementary pathways: (i) increased the mitochondrial size and improved the mitochondrial cristae organization; (ii) stimulated mitochondrial FA oxidation; (iii) reduced basal and maximal respiration and ATP production in SH cells; (iv) stimulated ATP production in SS cells; and (v) rescued the FA-induced apoptotic signals and oxidative stress in SH cells. We provide new insights about the direct protective effects of the nutraceutic silybin on hepatocytes mimicking *in vitro* NAFLD progression.

## Introduction

Hepatic steatosis is defined as the accumulation of triglycerides (TGs) exceeding 5% of liver weight. Without excess alcohol consumption, the simple steatosis (SS) is named non-alcoholic fatty liver, which may progress to more severe conditions, such as non-alcoholic fatty liver disease (NAFLD), non-alcoholic steatohepatitis (NASH) with hepatocyte injury and lobular and portal inflammation, and non-alcoholic liver cirrhosis with massive fibrosis and vascular remodeling (1), up to hepatocellular carcinoma (2, 3). Patients with NAFLD exhibit peculiar metabolic abnormalities, which include insulin resistance (IR), overweight, and obesity (4). Following the epidemics of obesity, NAFLD has become the leading cause of liver disease in developed countries.

Excess TG deposition in the liver occurs when hepatic availability of fatty acids (FAs) from plasma and/or *de novo* synthesis exceeds hepatic FA disposal by oxidation and TG export as very low-density lipoprotein (5). TG synthesis is an adaptive, beneficial response in hepatocytes exposed to potentially toxic concentrations of FAs or their metabolites. FAs and cholesterol, especially when accumulated in the mitochondria, are “aggressive” lipids leading to tumor necrosis factor α (TNFα) and reactive oxygen species (ROS) production and acting as early “inflammatory” hits (6), which contribute to promote NASH (7).

Excess TGs are stored in lipid droplets (LDs), a protective mechanism against FA lipotoxicity. LDs consist of a core of neutral lipids covered by phospholipids and proteins. Adipose differentiation-related protein (ADRP) is the major LD-associated protein, which promotes FA uptake and LD formation (8). In fact, the absence of ADRP reduces LD formation and protects against the development of steatosis (9). LDs also regulate lipid metabolism and trafficking through a network of molecules localized at the LD surface including the adipose triglyceride lipase (ATGL), which catalyzes the first reaction of TG hydrolysis (10). The master regulators of hepatic lipid metabolism are the peroxisome proliferator-activated receptors (PPARs), a family of transcription factors controlling both lipogenic and lipolytic pathways (11). FAs are endogenous ligands of all PPAR isofoms (12); uptake and oxidation of FAs are regulated mainly by PPARα, while their esterification and conversion to TGs by PPARγ whose expression typically increases in NAFLD (13).

Mitochondria are the main site for β-oxidation of FAs, which is primarily regulated by carnitine palmitoyltransferase 1 (CPT-1), an enzyme required for transport of long-chain FAs inside mitochondria (14). Mitochondria play an important role in the pathogenesis and progression of NAFLD, which has been proposed as a mitochondrial disease (15). Mitochondrial defects may underlie NAFLD process by altering energetic homeostasis and stimulating ROS production with consequent oxidative stress and impairment of fat oxidation processes (16). In addition to mitochondria, FAs are oxidized in peroxisomes by acyl-CoA oxidase (AOX) and in endoplasmic reticulum (ER) by CYP2E1; both processes lead to the overproduction of ROS and consequent oxidative stress, which triggers progression of steatosis toward NASH (17, 18).

Moreover, excess lipid accumulation disturbs the ER function, thereby generating ER stress. In the ER, we find the IκB kinase β (IKBKB)-interacting protein (IkBip), a serine kinase that plays a role in the nuclear factor kappa-B (NF-κB) signaling, which is activated by multiple stimuli such as inflammatory cytokines, DNA damages, and other cellular stresses (19). NF-kB activation was observed in the livers of obese mice where it causes liver inflammation and apoptosis and increases local and circulating levels of interleukins (20). In contrast, IkBip is associated with the progression of SS to steatohepatitis (SH) as it links steatosis with the activation of apoptotic pathways (21).

While the knowledge of the complex pathophysiological mechanisms involved in the onset and progression of livers steatosis has increased exponentially, a definitive therapy is still missing in NAFLD/NASH patients. Medicinal plants are largely employed as the source of dietary supplements (22). Silymarin, the extract from milk thistle (*Silybum marianum*), and its major active compound silybin are hepatoprotective agents for the treatment of liver diseases (23). Previous clinical findings evidenced the efficacy of silybin on improving IR and liver injury in patients with NAFLD (24) and in lowering some hepatic enzymes in patients with NASH (25). Moreover, silybin improved liver histology in a multicenter randomized controlled trial (26). However, the direct hepatoprotective activity of silybin in both non-alcoholic steatosis and SH remains to be elucidated and the possible mechanisms involving a potential anti-inflammatory effect require deeper investigations.

The present study aimed to clarify whether silybin may favorably affect lipid and radical homeostasis in *in vitro* model of NAFLD progression induced by sequential exposure of hepatoma FaO cells to high concentrations of FAs and TNFα. The experimental protocol mimics what occurs *in vivo* where chronically elevated levels of FAs induce IR in many organs and fat accumulation in the liver. In the adipose tissue, IR enhances lipolysis and increases the delivery of adipose-derived FAs to the liver. In contrast, in adipocytes FA excess increases TNFα production, which act on the liver by promoting inflammation and oxidative stress (6). Therefore, TNFα is the first inflammatory molecule linked with IR (27) and high serum levels of TNFα have been found in patients with NASH compared with healthy subjects (28). TNFα plasma levels correlate positively with the grade of liver fibrosis assessed by ultrasound-guided liver biopsy in patients with advanced stages of NAFLD (29). For this reason, we choose TNFα as the second hit to model *in vitro* NAFLD progression.

Our results show that silybin reduced excess TG accumulation in the *in vitro* models of both steatosis and SH. Changes in the expression of transcription factors such as PPARs and of enzymes for mitochondrial, peroxisomial, and ER oxidations of FAs have been evidenced. In particular, silybin rescued the FA-induced mitochondrial dysfunction as well as apoptotic signals and oxidative stress characteristic of SH condition.

## Materials and Methods

### Cell Treatments

Rat hepatoma FaO cells (European Collection of Cell Cultures, Sigma-Aldrich Corp.) (30) were grown in Coon’s modified Ham’s F12 with 10% fetal bovine serum. Cells were grown until 80% confluence, incubated in high-glucose medium with 0.25% bovine serum albumin (BSA) to increase stability and solubility of FFA (31). A condition mimicking human steatosis (SS) was induced by incubating FaO cells for 3 h with oleate/palmitate mixture (2:1 M ratio, final concentration 0.75 mM). A SH condition was mimicked by incubating SS cells for 24 h with 10 ng/mL TNFα (32). After replacing the medium, both SS and SH cells were treated for 24 h with 50 µM silybin (S) [Istituto Biochimico Italiano (IBI), Lorenzini, Italy]. Silybin stock (10 mM) was prepared in dimethyl sulfoxide.

### Cell Viability and Apoptosis

For both resazurin and sulforhodamine B (SRB) assays 1.5 × 10^4^ cells/well were seeded in 96-well plates. For resazurin assay, after treatment, the medium was removed and cells were incubated for 30 min with medium supplemented with 10 µg/mL resazurin. The reduction of resazurin to resorufin, indicative of metabolic activity, was measured fluorimetrically (λ_exc_ 570 nm; λ_em_ 600 nm) in Biotek-Cytation 3 reader (Biotek Instruments, Winooski, VT, USA) (33). For SRB assay, cells were fixed with ice-cold methanol containing 1% acetic acid for 30 min and then incubated with 0.5% SRB in 1% acetic acid for 1 h at 37°C. The unbound dye was removed with 1% acetic acid solution. The dye bound to proteins was extracted with 10 mM Tris-base solution pH 10, and absorbance was measured at 540 nm (34).

For apoptosis assessment, cells were collected and the pellet resuspended in 20 mM HEPES/NaOH pH 7.5, 250 mM sucrose, 10 mM KCl, 2 mM MgCl_2_, 1 mM EDTA, 2 mM dithiothreitol (DTT), and 100 µM phenylmethylsulfonyl fluoride (PMSF), a protease-inhibitor cocktail (1 µg/mL of leupeptin, antipain, chymostatin, and pepstatin A). Caspase 3-like activity was measured in cell extracts containing 25 µg protein determined by the bicinchoninic acid method using BSA as a standard (35). For calibration, known concentrations of p-nitroanilide (pNA) were measured. Cell extracts were incubated for 1 h at 37°C in 25 mM Hepes pH 7.5, 10% sucrose, 10 mM DTT, 0.1% CHAPS, and 100 µM caspase substrate Ac-DEVD-pNA; the pNA released after cleavage from the substrate was measured. The results are expressed as amount of nanomoles of pNA released per microgram of protein (36).

### TG Content

Cells were scraped and lysed. Lipids were extracted using the chloroform/methanol (2:1) method (37). TG content was quantified spectrophotometrically by using “Triglycerides liquid” kit (Sentinel, Milan, Italy) in a Varian Cary-50Bio spectrophotometer (Agilent, Milan, Italy). Values were normalized to protein content. Data are expressed as percent TG content relative to controls.

### LD Imaging

Cells grown on coverslips were rinsed with phosphate-buffered saline (PBS) pH 7.4 and fixed with 4% paraformaldehyde for 20 min at room temperature. Slides were incubated with 1 µg/mL BODIPY 493/503 (Molecular Probes, Life technologies, Monza, Italy) in PBS for 30 min (38). After washing and mounting with 4′,6-diamidino-2-phenylindole (DAPI) (ProLong Gold medium with DAPI; Invitrogen) slides were examined by Nikon Eclipse E80i light microscope (Nikon, Tokyo, Japan) equipped with the standard epifluorescence filter set up. Images were captured under oil with a 100× plan apochromat objective. Analyses were performed on two independent experiments measuring at least 40 cells for each treatment using the ImageJ software.^1^

### Mitochondria Imaging

Cells grown on μ-slides eight-well ibiTreat (Ibidi, Germany) were incubated with 50 nM tetramethylrhodamine ethyl ester (TMRE) and 1 µg/µL Hoechst for 30 min at 37°C in the dark. A lower concentration of TMRM^+^ was maintained in the medium to avoid leakage from mitochondria. Cells were observed under a Nikon Eclipse Ti-S microscope. Images were obtained through LSM Image Browser (39).

### Electron Microscopy

Cells grown on glass chamber slides (Lab-Tek, Nunc, 177380) were washed in 0.1 M cacodylate buffer and fixed in 0.1 M cacodylate buffer containing 2.5% glutaraldehyde for 1 h at room temperature. Cells were post-fixed in osmium tetroxide for 2 h and 1% uranyl acetate for 1 h. Samples were dehydrated through a graded ethanol series and flat embedded in resin (Poly-Bed; Polysciences, Inc., Warrington, PA, USA) for 24 h at 60°C. Ultrathin sections (50 nm) were cut parallel to the substrate until reaching the apical surface, stained with 5% uranyl acetate in 50% ethanol, and observed with CM10 electron microscope (Philips, Eindhoven, the Netherlands), images were taken with a Megaview 3 camera. Mitochondrial number and size were assessed in 12 randomly taken images at 25k magnification for each treatment. The mitochondrial length (major axis) and width (minor axis) were measured with iTEM software package (Olympus-SYS). Statistical analysis for mitochondrial diameter was performed using the Mann–Whitney test. The total number of mitochondria and mitochondrial cristae was manually scored. Statistical analysis was performed with Student’s *t*-test.

### Catalase Activity

Catalase activity was evaluated spectrophotometrically in both 12,000 × *g* of supernatant and pellet of hepatocyte lysates (40). Catalase specific activity was expressed as micromoles of decomposed H_2_O_2_ per minute per milligram of sample protein. Data are expressed as percent relative to controls.

### Lipid Peroxidation

Lipid peroxidation was determined through the thiobarbituric acid reactive substances assay based on the reaction of malondialdehyde (MDA; 1,1,3,3-tetramethoxypropane) with thiobarbituric acid (TBA) (41). Briefly, 1 vol of cell suspension was incubated for 45 min at 95°C with 2 vol of TBA solution (0.375% TBA, 15% trichloroacetic acid, 0.25 N HCl) and, then, 1 vol of *N*-butanol was added and the absorbance of the organic phase was measured spectrophotometrically. Results were expressed as picomoles MDA per milliliter/milligram protein. Data are expressed as percent relative to controls.

### 8-Hydroxy-2-Deoxy Guanosine (8-OHdG) Release

The 8-OHdG excreted into the medium after DNA repair/turnover was quantified using DNA/RNA Oxidative Damage ELISA kit (Cayman Chemical Company, MI, USA). Samples were analyzed in duplicate. Standard 8-OHdG was assayed over a concentration range of 10.3–3,000 pg/mL (42). Results were expressed as picogram 8-OHdG per milliliter.

### RNA Extraction and Real-time Quantitative PCR (qPCR)

RNA was isolated using Trizol reagent, cDNA was synthesized, and real-time qPCR performed in quadruplicate using 1× IQ™SybrGreen SuperMix and Chromo4™ System apparatus (Bio-Rad, Milan, Italy) (43). The relative quantity of target mRNA was calculated by the comparative Cq method using glyceraldehyde 3-phosphate dehydrogenase (GAPDH) as housekeeping gene and expressed as fold induction with respect to controls (44). Primer pairs designed *ad hoc* starting from the coding sequences of *Rattus norvegicus*^2^ and synthesized by TibMolBiol (Genova, Italy) are listed in Table 1.

**Table 1:** Primer sequences table.

Primer name	Primer sequence (5′→3′)	Annealing temperature (°C)	Accession ID
Adipose differentiation-related protein (ADRP) Fwd	CCGAGCGTGGTGACGAGGG	64	AAH85861
ADRP Rev	GAGGTCACGGTCCTCACTCCC		
Glyceraldehyde 3-phosphate dehydrogenase (GAPDH) Fwd	GACCCCTTCATTGACCTCAAC	60	DQ403053
GAPDH Rev	CGCTCCTGGGAAGATGGTGATGGG		
IκB kinase β-interacting protein (IkBip) Fwd	CAGAACAGTGAGCAGGCAAG	60	NM_001009430.2
IkBip Rev	ACGGCATTCTCTATGGTTGG		
Peroxisome proliferator-activated receptor (PPAR)α Fwd	CCCCACTTGAAGCAGATGACC	60	NM_013196
PPARα Rev	CCCTAAGTACTGGTAGTCCGC		
PPARγ Fwd	CGGAGTCCTCCCAGCTGTTCGCC	60	Y12882
PPARγ Rev	GGCTCATATCTGTCTCCGTCTTC		
PPARδ Fwd	AATGCCTACCTGAAAAACTTCAAC	60	AJ306400.1
PPARδRev	TGCCTGCCACAGCGTCTCAAT		
Carnitine palmitoyltransferase 1 (CPT-1) Fwd	CCGCTCATGGTCAACAGCA	60	NM_031559
CPT-1 Rev	CAGCAGTATGGCGTGGATGG		
CYP2E1 Fwd	ACCTTCAGTCACTGGACATCAA	60	BC081774
CYP2E1 Rev	AGGATCAGGAGCCCATATCTC		
Adipose triglyceride lipase (ATGL) Fwd	CGGTGGATGAAGGAGCAGACA	60	NM_001108509
ATGL Rev	TGGCACAGACGGCAGAGACT		
APE1 Fwd	CAGATCAGAAAACGTCAGCCAG	60	NM_024148.1
APE1 Rev	GGTCTCTTGGAGGCACAAGATG		
POLG Fwd	GAAGAGCGTTACTCTTGGACCAG	62	NM_053528.1
POLG Rev	AACATTGTGCCCCACCACTAAC		
COII Fwd	TGAGCCATCCCTTCACTAGG	60	X14848.1
COII Rev	TGAGCCGCAAATTTCAGAG		
β-Actin Fwd	CTGCTCTTTCCCAGATGAGG	60	NM_031144.2
β-Actin Rev	CCACAGCACTGTAGGGGTTT		

### mtDNA Copy Number

DNA extracted using Genomic-tip 20/G kit (Qiagen, Valencia, CA, USA) was quantified using the PicoGreen dsDNA reagent (Invitrogen, Milan, Italy). Relative mtDNA copy numbers were measured by qPCR using iQ5 System Apparatus (Bio-Rad) and corrected by simultaneous measurement of nuclear DNA. Amplification of mitochondrial cytochrome *c* oxidase subunit II (COII, mitochondrial-encoded gene) and β-actin (nuclear-encoded gene) was performed using the primers listed in Table 1. The mtDNA content was calculated using ΔCt = average Ctnuclear DNA − average CtmtDNA and, then, was obtained using the formula mtDNA content = 2(2ΔCt).

### Western Blotting

In nuclear homogenates, NF-kB/p65 was detected by Western blot. Cellular pellet was suspended in 400 µL ice-cold Buffer A (20 mM Tris-HCl pH 7.8, 50 mM KCl, 10 µg/mL Leupeptin, 0.1 mM DTT, 1 mM PMSF) and 400 µL Buffer B (Buffer A plus 1.2% Non-indet P40). After vortex and centrifugation (14,000 × *g* for 30 s, 4°C) the nuclear pellet was washed with 400 µL Buffer A, resuspended in 100 µL Buffer B, mixed in ice for 15 min, and centrifuged (14,000 × *g* for 20 min, 4°C). The supernatant containing nuclei was collected, and 30–50 µg proteins were electrophoresed on SDS polyacrylamide gel electrophoresis (45). Membrane was blocked for 1 h in 5% fat-free milk/PBS (pH 7.4), probed overnight using rabbit anti-human NF-kB p65 (SC-109; Santa Cruz Biotechnology, DBA, Milan, Italy) in PBST buffer (PBS 0.1% Tween 20) at 4°C (46), and then washed and incubated with horseradish peroxidase-conjugated rabbit anti-mouse IgG (Sigma-Aldrich) in PBST for 1 h at room temperature. Immune complexes were visualized using chemiluminescence western blotting analysis system (Bio-Rad ChemiDoc XRS System). Films were digitized and band optical densities were quantified against the actin band and expressed as Relative Optical Density (ROD, arbitrary units). ROD of each band was expressed as percentage respect to control.

### Oxygen Consumption

Oxygen consumption was measured at 37°C using Seahorse XFe96 Extracellular Flux Analyzer (Seahorse Bioscience, Germany). About 2 × 10^4^ cells/well were seeded in 96-well plates. In parallel, an XFe96 sensor cartridge for each cell plate was placed in a 96-well calibration plate containing 200 µL/well calibration buffer and left to hydrate overnight at 37°C. Medium was replaced with 200 µL/well of low-buffered serum-free medium pH 7.4 left at 37°C for 1 h without CO_2_ to allow de-gassing the plate. A final concentration of 3 µM oligomycin, 1 µM carbonyl cyanide-4-(trifluoromethoxy)phenylhydrazone (FCCP), and a mixture of 1 µM rotenone and 1 µM antimycin were added sequentially to cells from prot A, B, and C, respectively. A total of 25 µM of each compound was pre-loaded into the ports of each well in the XFe96 sensor cartridge. The sensor cartridge and the calibration plate were loaded into the XFe96 Extracellular Flux Analyzer for calibration, and then, the calibration plate was replaced with the study plate. Three baseline rate measurements of oxygen consumption rate (OCR) were made using a 3 min mix and 3 min measure cycle. The compounds were injected pneumatically by the XFe96-Analyzer into each well, mixed, OCR measurements made using the 3 min mix and 3 min measure cycle (47).

### Statistical Analysis

Data are expressed as mean ± SD of at least three independent experiments in triplicate. Statistical analysis was performed using ANOVA with Tukey’s post-test (GraphPad Software, Inc., San Diego, CA, USA).

## Results

### Effects of Silybin on Intracellular Fat Overload

**Figure 1:**
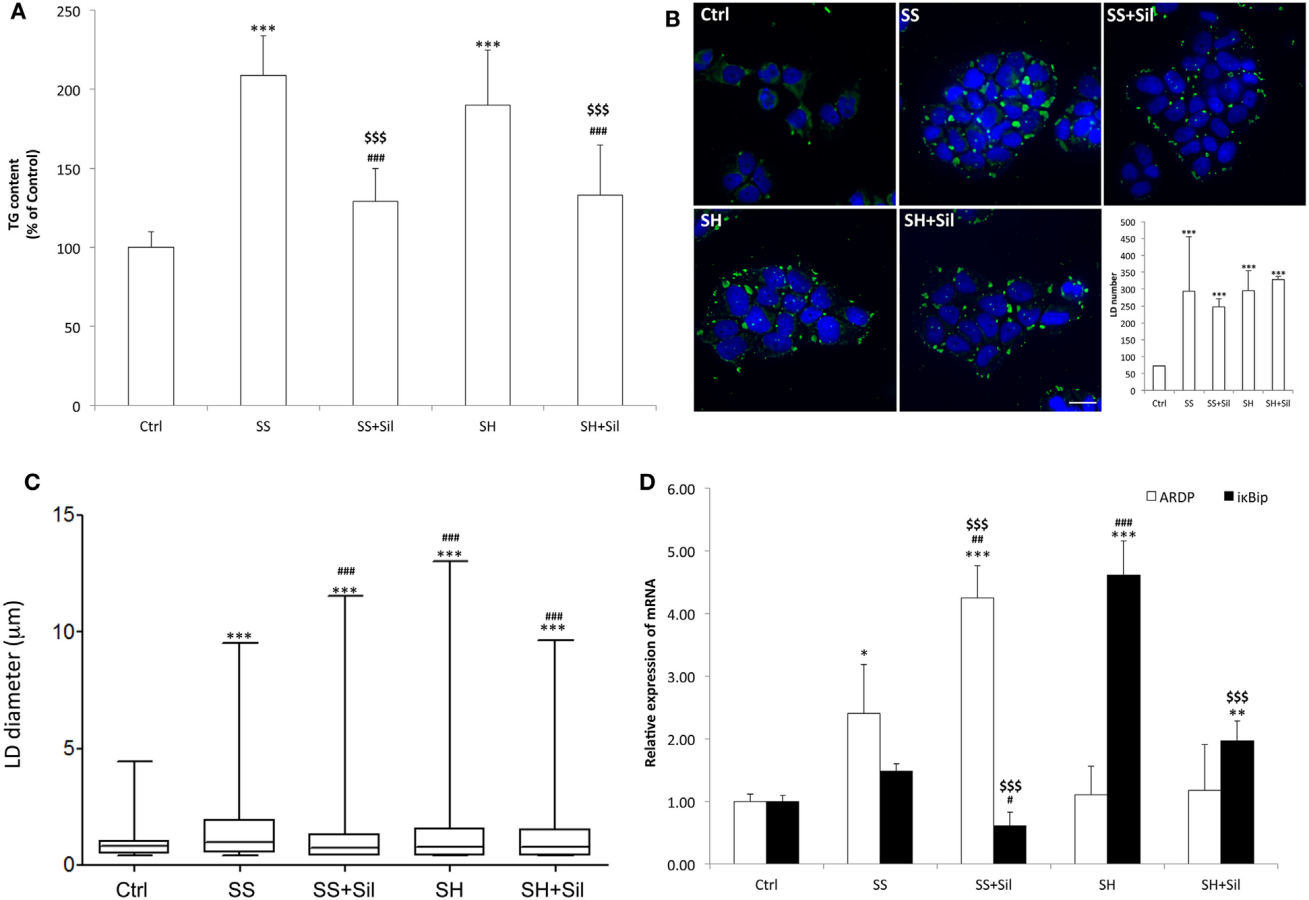
Effect of silybin on lipid accumulation. Lipid accumulation parameters were measured in FaO cells incubated in the absence (Ctrl) or in the presence of fatty acids [simple steatosis (SS)] or fatty acids and tumor necrosis factor α (TNFα) [steatohepatitis (SH)] and subsequently treated with 50 µM silybin (SS + Sil or SH + Sil, respectively). **(A)** Intracellular triglyceride (TG) content quantified by spectrophotometric assay. Data are expressed as percent TG content relative to control and normalized for total proteins determined with bicinchoninic acid (BCA). Values are mean ± SD from at least three independent experiments. **(B)** Lipid droplets (LDs) visualized by fluorescence microscopy using BODIPY 493/503 (green fluorescence) and nuclei visualized by 4′,6-diamidino-2-phenylindole (DAPI) (blue fluorescence) (magnification 100×; scale bar: 10 µm). Representative images and the average LD number for all the experimental conditions were also shown. **(C)** Average LD size measured on two independent experiments (40 cells per treatment for each experiment) by using Huygens professional suite software. The results were plotted as box-and-whisker plot, showing the interquartile range, the median as horizontal bar and the whiskers are the minimum and maximum values. **(D)** The mRNA expression of adipose differentiation-related protein (ADRP) and IκB kinase β-interacting protein (IkBip) evaluated by quantitative PCR (qPCR); glyceraldehyde 3-phosphate dehydrogenase (GAPDH) was used as the internal control and data expressed as fold induction with respect to controls. ANOVA followed by Tukey’s test was used to assess the statistical significance between groups. Significant differences are denoted by symbols: Ctrl vs all treatments ***p ≤ 0.001, **p ≤ 0.01, *p ≤ 0.05; simple steatosis (SS) vs all treatments ^###^*p* ≤ 0.001, ^##^*p* ≤ 0.01, ^#^*p* ≤ 0.05; steatohepatitis (SH) vs all treatments ^$$$^*p* ≤ 0.001.

Intracellular TG content increased significantly and to a similar extent in steatotic (SS) (+109%; *p* ≤ 0.001) and in SH (+90%; *p* ≤ 0.001) cells compared to control (Figure 1A). Exposure to silybin 50 µM did not affect the TG content of control cells (data not shown) but significantly decreased to a similar extent the TG content in both SS and SH cells (−38 and −30%, respectively; *p* ≤ 0.001 *vs* their counterpart).

Lipid accumulation was associated to increased number and size of LDs, as visualized by BODIPY staining. At fluorescence microscopy, control cells showed few (about 72 LDs/cell) and small (0.9 µm average diameter) LDs, which were dispersed in the cytosol (Figures 1B,C). In SS, we observed an increase in both number (294 LDs/cell) and size (1.5 µm) of LDs, with average diameter increasing of about +67% (*p* ≤ 0.001) compared to controls, and sylibin reduced the LD diameter to a value similar to controls (1.1 µm; *p* ≤ 0.001) (Figures 1B,C). In contrast, SH cells displayed a LD number similar to that of SS cells but a LD size slightly lower (1.3 µm) (Figures 1B,C); silybin did not affect LD size.

The mRNA levels of ADRP and IkBip, two markers of LDs and NAFLD progression, respectively, were analyzed by qPCR (Figure 1D). Excess lipid accumulation in SS cells resulted in up-regulation of ADRP expression (2.4-fold induction vs control; *p* ≤ 0.05) that was further stimulated by silybin (4.2-fold induction compared to control; +77% vs SS; *p* ≤ 0.01). In SH cells, ADRP expression was at a level similar to control and it was unaffected by silybin. In regard to IkBip expression, we observed only a small, non-significant up-regulation in SS cells, while it was dramatically up-regulate in SH cells (4.6-fold induction compared to control; *p* ≤ 0.001). Exposure to silybin greatly and comparably reduced IkBip expression in both SS (−59% with respect to SS; *p* ≤ 0.05) and SH (−57% with respect to SH; *p* ≤ 0.001) cells. Sylibin did not affect ADRP and IkBip expression in control cells (data not shown).

### Effects of Silybin on Lipid Metabolism

**Figure 2:**
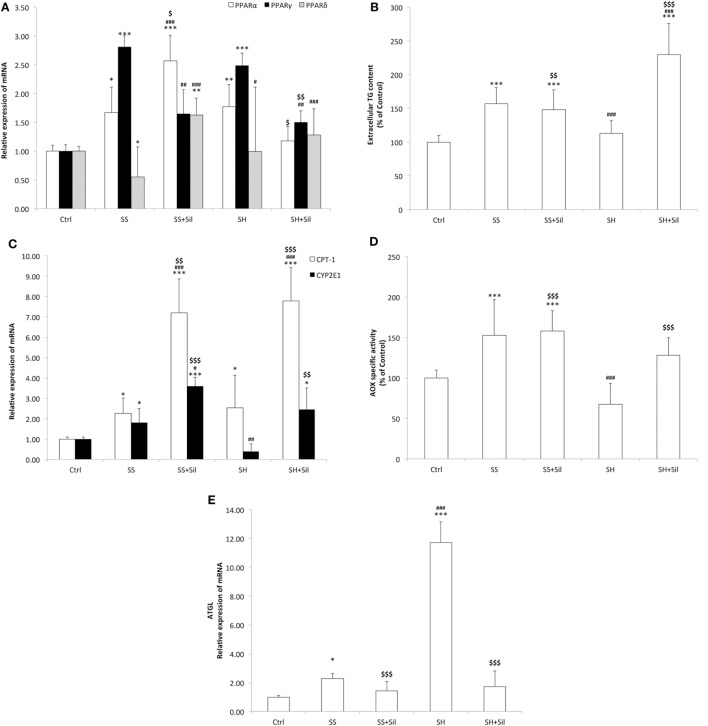
Effect of Silybin on lipid metabolism. In FaO cells treated as described above the following parameters were evaluated: **(A)** The mRNA expression of peroxisome proliferator-activated receptor (PPAR) α, PPARγ, and PPARδ by quantitative PCR (qPCR). **(B)** The extracellular triglyceride (TG) content by spectrophotometric analysis. **(C)** The mRNA expression of carnitine palmitoyltransferase 1 (CPT-1) and CYP2E1 by qPCR. **(D)** The enzymatic activity of peroxisomial acyl CoA oxidase (AOX) (nmol H_2_O_2_/min/mg protein) by using spectrophotometric assay. Data are expressed as percentage values with respect to controls and normalized for total proteins. **(E)** The mRNA expression of adipose triglyceride lipase (ATGL) by quantitative PCR (qPCR). For qPCR experiments, glyceraldehyde 3-phosphate dehydrogenase (GAPDH) was used as the internal control and data were expressed as fold induction with respect to controls. Values are mean ± SD from at least three independent experiments. ANOVA followed by Tukey’s test was used to assess the statistical significance between groups. Significant differences are denoted by symbols: Ctrl vs all treatments ***p ≤ 0.001, **p ≤ 0.01, *p ≤ 0.05; simple steatosis (SS) vs all treatments ^###^*p* ≤ 0.001, ^##^*p* ≤ 0.01, ^#^*p* ≤ 0.05; steatohepatitis (SH) vs all treatments ^$$$^*p* ≤ 0.001, ^$$^*p* ≤ 0.01, and ^$^*p* ≤ 0.05.

Lipid metabolism is regulated by PPARs, the main transcription factors for lipolytic and lipogenic genes (48). The mRNA level of PPARs was assessed by qPCR (Figure 2A). As compared to control cells, SS cells showed a significant increase in expression of PPARα and PPARγ (1.29-fold induction, *p* ≤ 0.05; 2.8-fold induction, *p* ≤ 0.001, respectively), and a down-regulation of PPARδ (about 0.55-fold induction; *p* ≤ 0.05). The lipid-lowering action of silybin was sustained by increased expression of PPARα (2.6-fold induction compared to controls; +99% compared to SS, *p* ≤ 0.01), decreased expression of PPARγ (1.6-fold induction compared to controls, −42% compared to SS; *p* ≤ 0.05), and increased expression of PPARδ (1.6-fold induction compared to controls; +193% compared to SS, *p* ≤ 0.001). In SH hepatocytes, the profile of PPARα and PPARγ expression was similar to SS, whereas PPARδ expression showed values similar to controls. Exposure of SH cells to silybin reduced transcription of both PPARα (1.1-fold induction compared to controls; −39% compared to SH, *p* ≤ 0.05) and PPARγ (1.5-fold induction compared to controls; −48% compared to SH, *p* ≤ 0.01), without affecting PPARδ expression.

The lipid-lowering action of silybin could be sustained by increased TG secretion, since SS cells released more TGs into culture medium than controls (+157%; *p* ≤ 0.001). The addition of silybin 50 µM did not modify this picture. In SH cells, a significant reduction in TG secretion was observed compared to SS (−28%; *p* ≤ 0.001). The addition of sylibin counteracted this condition as TG secretion was stimulated (+103% with respect to SH; *p* ≤ 0.001) (Figure 2B).

In contrast, TG accumulation might be also reduced by stimulation of oxidative pathways taking place in mitochondria, peroxisomes, and ER (43). Thus, mitochondrial CPT-1 and peroxisomal AOX for β-oxidation and microsomal cytochrome P450 (CYP) 2E1 for ω-oxidation were assessed. The mRNA levels of CPT-1 were significantly up-regulated in both SS and SH cells (2.44- and 2.91-fold inductions, respectively; *p* ≤ 0.05) and further increased upon incubation with silybin (+199% and +164 with respect to SS and SH cells, respectively; *p* ≤ 0.001) (Figure 2C). CYP2E1 expression was up-regulated in SS cells (2.20-fold induction; *p* ≤ 0.001) and was further increased upon incubation with silybin (+63% with respect to SS; *p* ≤ 0.001), whereas in SH hepatocytes, CYP2E1 expression was at basal level and it was markedly increased by silybin (+518 with respect to SH cells; *p* ≤ 0.01) (Figure 2C). In peroxisomes, AOX activity was stimulated in SS cells (+53%, *p* < 0.001 compared to controls), while silybin did not change it. We observed a decrease in AOX activity in SH cells compared to SS (−56% with respect to SS; *p* < 0.001) and an increase upon silybin treatment (+90% with respect to SH cells; *p* < 0.001) (Figure 2D).

Up-stream of FA oxidation there is ATGL, a lipase controlling lipid mobilization from LDs. A significant increase in ATGL transcripts was observed in SS cells (2.29-fold induction with respect to control; *p* ≤ 0.05) and a larger increase in SH cells (11.7-fold induction with respect to controls; *p* ≤ 0.001) (Figure 2E). ATGL mRNA expression was reduced after incubation of SH cells with silybin (−85% compared to SH; *p* ≤ 0.001).

### Hepatoprotective Effects of Silybin

**Figure 3:**
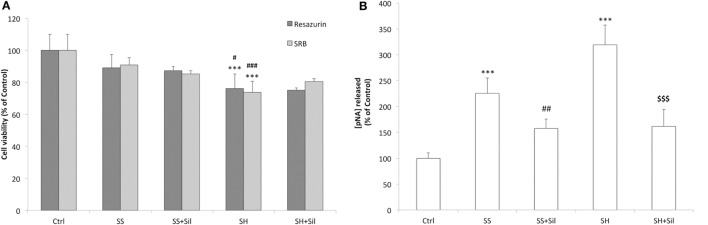
Effect of silybin on cell proliferation and apoptosis. Under the conditions of Figure 1, the following parameters were measured: **(A)** The metabolic activity and the cell mass by resazurin and sulforhodamine B (SRB) assay, respectively. Data are expressed as percentage values with respect to controls. **(B)** The activity of effector caspase 3, measured as p-nitroanilide (pNA) released after cleavage from the substrate (nmoles of pNA released/μg protein). Calibration was done using known concentrations of pNA. Values are mean ± SD from at least three independent experiments. ANOVA followed by Tukey’s test was used to assess the statistical significance between groups. Significant differences are denoted by symbols: Ctrl vs all treatments ***p ≤ 0.001; simple steatosis (SS) vs all treatments ^###^*p* ≤ 0.001, ^##^*p* ≤ 0.01, ^#^*p* ≤ 0.05; steatohepatitis (SH) vs all treatments ^$$$^*p* ≤ 0.001.

We observed no significant changes in cell viability in SS cells, whereas a reduced viability was observed in SH cells (−24% compared to control; *p* ≤ 0.001) and silybin did not counteract this effect (Figure 3A). Similar results were supplied by cell density analysis: no significant decrease in cell density occurred in SS cells, while cell density was reduced in SH cells (−26% compared to control; *p* ≤ 0.001) and silybin did not counteract this effect (Figure 3A). In regard to apoptosis, we observed an increase in the caspase 3-like activity in SS cells (+126% compared to control; *p* ≤ 0.001) and a larger increase in SH cells (+219% compared to control; *p* ≤ 0.001). Of note, incubation of both SS and SH cells with silybin significantly reduced caspase 3-like activity (−68 and 158%, respectively, in SS + sil and SH + sil; *p* ≤ 0.01 and *p* ≤ 0.001) (Figure 3B).

### Effects of Silybin on Mitochondrial Activity and Damage

**Figure 4:**
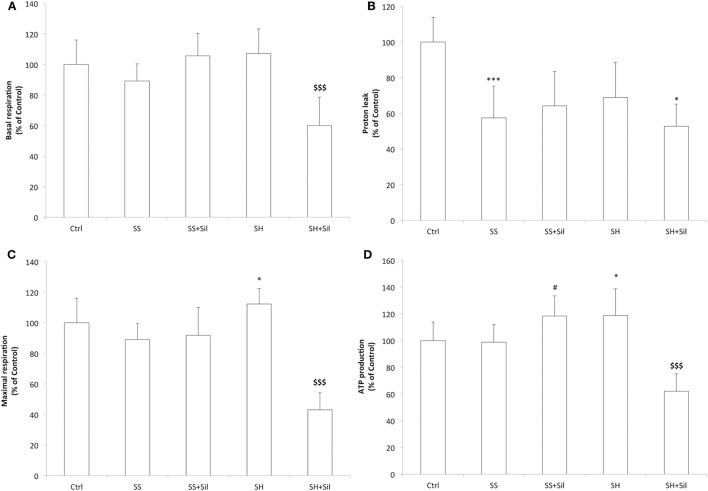
Effect of silybin on mitochondrial oxygen consumption rates (OCRs). Under the conditions of Figure 1, we measured the cellular OCR by using Seahorse XFe96 Extracellular Flux Analyzer. The following respiratory parameters were evaluated: **(A)** Cell basal respiration (OCR pmol/min/SRB labeling). **(B)** Proton leak (OCR pmol/min/SRB labeling). **(C)** Cell maximal respiration (OCR pmol/min/SRB labeling); **(D)** ATP production (OCR pmol/min/SRB labeling). Data are expressed as mean ± SD of 14 separate experiments. ANOVA followed by Tukey’s test was used to assess the statistical significance between groups. Significant differences are denoted by symbols: Ctrl vs all treatments ***p ≤ 0.001, *p ≤ 0.05; SS vs all treatments, ^#^*p* ≤ 0.05; SH vs all treatments ^$$$^*p* ≤ 0.001.

Mitochondrial respiration was analyzed by using the Seahorse Extracellular Flux Analyzer (Figure 4). Basal respiration remained unchanged across control, SS, and SH cells; silybin did not affect basal respiration of SS cells, but significantly reduced that of SH cells (−47% with respect to control; *p* ≤ 0.001) (Figure 4A). The proton leak was significantly reduced in both SS and SH cells with respect to controls (−42% and −31%, respectively; *p* ≤ 0.001 and *p* ≤ 0.05), and silybin did not influence this picture (Figure 4B). Maximal respiration remained unchanged between control, SS cells, and SS cells exposed to sylibin, but it was significantly increased in SH cells (+12% respect to control, *p* ≤ 0.05) and silybin dramatically reduced it (−70%, compared to SH; *p* ≤ 0.001) (Figure 4C). Also, the oxygen consumption associated with ATP production did not change in SS cells, while it increased in SH cells (+20% respect to control; *p* ≤ 0.05). Silybin exerted an opposite effect by increasing ATP production in SS cells (+20% respect to SS; *p* ≤ 0.05) and decreasing it in SH cells (−56% respect to SH cells; *p* ≤ 0.001) (Figure 4D).

**Figure 5:**
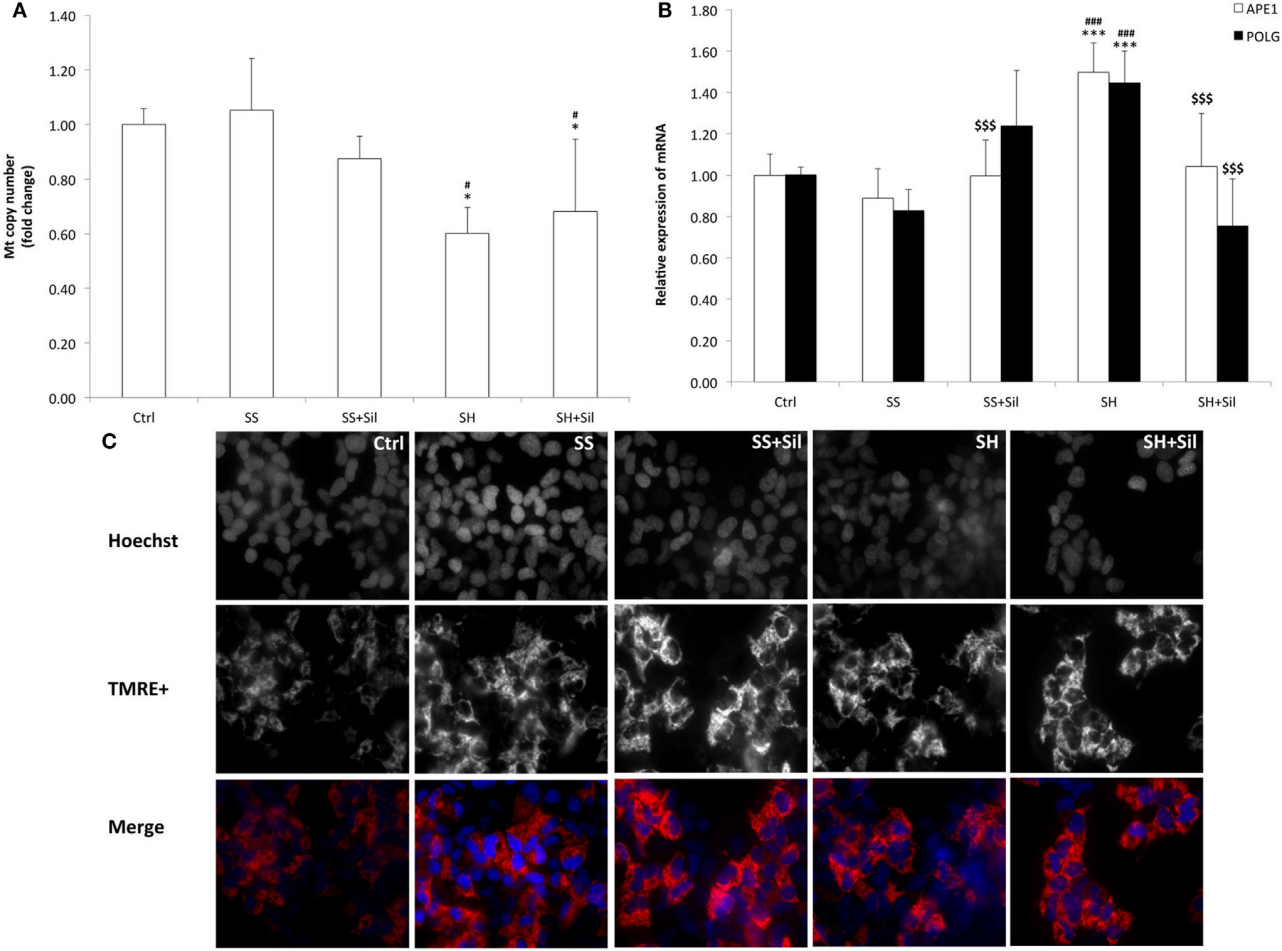
Effect of silybin on mitochondrial DNA (mtDNA) copy number and membrane polarization. Under the conditions of Figure 1, the following parameters were measured: **(A)** mtDNA copy number by quantitative PCR (qPCR) using primer for COII. The nuclear gene β-actin was used as the internal control; data were expressed as fold induction with respect to controls. **(B)** The mRNA expression of APE1 and POLG genes related to mtDNA repair; β-actin was used as the internal control and data were expressed as fold induction with respect to controls. **(C)** The mitochondrial polarization through cell vital staining with TMRE (mitochondria, red fluorescence) and Hoechst33342 (nuclei, blue fluorescence). Representative images for all the experimental conditions are reported separately for each staining (black/white) and then merged in colors. Images were acquired using 40× objective (scale bar 35 µm). Values are mean ± SD from at least three independent experiments. ANOVA followed by Tukey’s test was used to assess the statistical significance between groups. Significant differences are denoted by symbols: Ctrl vs all treatments ***p ≤ 0.001, *p ≤ 0.05; simple steatosis (SS) vs all treatments ^###^*p* ≤ 0.001, ^#^*p* ≤ 0.05; steatohepatitis (SH) vs all treatments ^$$$^*p* ≤ 0.001.

To assess possible mitochondria damage, we evaluated the mtDNA copy number and expression of the two main genes involved in mtDNA repair by qPCR (Figure 5). The mtDNA copy number did not change in SS cells, but it was reduced in SH cells (−43% respect to control; *p* ≤ 0.05), and silybin was not able to rescue this damage (Figure 5A). With regard to the enzymes for DNA repair, SH cells exhibited an increase in mRNA expression of both APE1 (1.50-fold induction compared to control; +68% with respect to SS, *p* ≤ 0.001) and POLG (1.45-fold induction compared to control; +75% with respect to control, *p* ≤ 0.001), and silybin counteracted this effect (−30% for APE1 and −48% for POLG respect to SH; *p* ≤ 0.001) (Figure 5B).

In contrast, fluorescence microscopy of TMRE-stained mitochondria was performed to assess the potential of mitochondrial inner membrane (Figure 5C). We observed a higher fluorescent signal in SS cells than in control cells, and fluorescence was reduced and less organized in SH cells where it appeared rather diffuse and irregular. Treatment of both SS and SH cells with silybin increased TMRE signal with cells appearing more brilliant compared to untreated counterparts.

**Figure 6:**
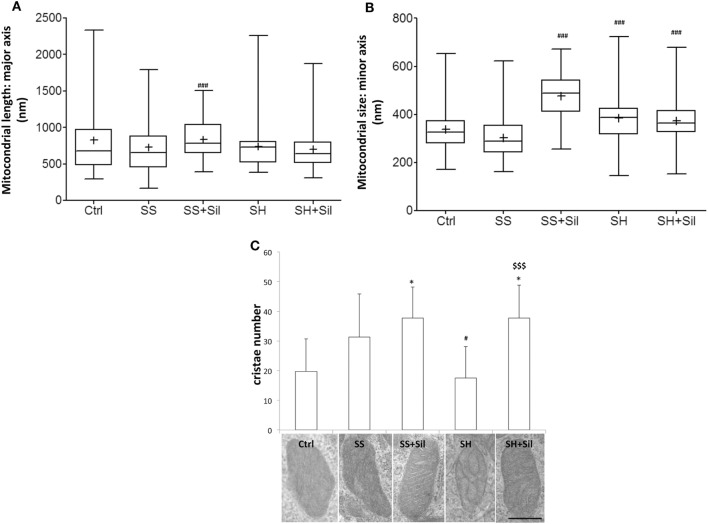
Effect of silybin on mitochondrial shape. Under the conditions of Figure 1, cells were analyzed by electron microscopy and the following parameters were assessed: **(A)** mitochondrial length (major axis) and mitochondrial size (minor axis) **(B)** were measured. The results were plotted as box-and-whisker plot, showing the interquartile range, the median as horizontal bar and the whiskers are the minimum and maximum values. **(C)** Cristae number was also evaluated. Values are mean ± SD from all mitochondria scored in 20 images for each experimental condition. Representative electron micrographs of randomly selected mitochondria from each experimental condition are also shown. (magnification 25k; scale bar: 0.5 µm). Significant differences are denoted by symbols: Ctrl vs all treatments, *p ≤ 0.05; simple steatosis (SS) vs all treatments ^###^*p* ≤ 0.001 and ^#^*p* ≤ 0.05; steatohepatitis (SH) vs all treatments ^$$$^*p* ≤ 0.001.

Ultrastructural morphology of mitochondria was assessed by EM analysis to measure mitochondrial number, major and minor axe diameters, number, and organization of cristae (Figures 6A–C). Our results showed that both mitochondrial morphometry and cristae number did not change in SS cells. Upon treatment of SS cells with silybin mitochondria showed a significant increase in size (both major and minor axes, *p* ≤ 0.001) and the mitochondrial cristae increased in number and were better organized and parallel to each other’s (Figures 6A–C). In SH cells, mitochondrial size did not change (Figures 6A,B) and the mitochondrial cristae were largely disorganized and significantly reduced in number compared to SS cells (*p* ≤ 0.05) (Figure 6C). Interestingly, in SH cells, treatment with silybin rescued both the number (*p* ≤ 0.001) and the parallel organization of mitochondrial cristae compared to SS cells (Figure 6C).

### Effects of Silybin on Oxidative Stress

**Figure 7:**
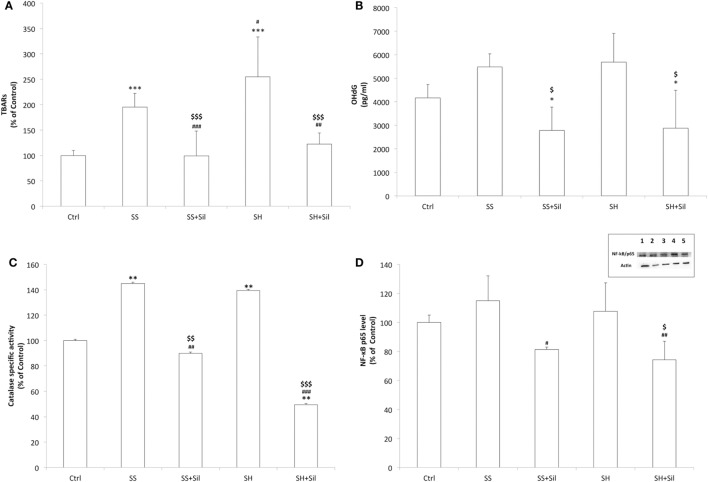
Effect of silybin on oxidative stress end-points. Oxidative stress end-points were determined in FaO cells treated under the conditions described in Figure 1: **(A)** the intracellular level of MDA (pmol MDA/mL/mg of proteins) by spectrophotometric thiobarbituric acid reactive substances (TBARS) assay. Data are expressed as percentage values with respect to controls and normalized for total proteins. **(B)** Levels of 8-hydroxy-2-deoxy guanosine (8-OHdG) (pg/mL) released into the culture medium measured by competitive ELISA. **(C)** Catalase enzymatic activity (μmoles of decomposed H_2_O_2_/min/mg of proteins) by spectrophotometric assay. Data are expressed as percentage values with respect to controls and normalized for total proteins. **(D)** Densitometric analysis of nuclear nuclear factor kappa-B (NF-κB)/p65 evaluated by Western blotting. Data are expressed as percentage values with respect to controls and normalized for β-actin. Immune complexes were visualized using an enhanced chemiluminescence, quantified using a computerized imaging system (Biorad Quantity One 1-D Analysis Software) and expressed as relative optical density (ROD, arbitrary units). Inset: representative image of NF-κB/p65 immunoreactive bands: control (lane 1), simple steatosis (SS) (lane 2), SS + Sil (lane 3), steatohepatitis (SH) (lane 4), and SH + Sil (lane 5).Values are mean ± SD from at least three independent experiments. ANOVA followed by Tukey’s test was used to assess the statistical significance between groups. Significant differences are denoted by symbols: Ctrl vs all treatments ***p ≤ 0.001, **p ≤ 0.01, *p ≤ 0.05; SS vs all treatments ^###^*p* ≤ 0.001, ^##^*p* ≤ 0.01, ^#^*p* ≤ 0.05; SH vs all treatments ^$$$^*p* ≤ 0.001, ^$$^*p* ≤ 0.01, ^$^*p* ≤ 0.05.

Increased fat oxidation produces excess of ROS, which react with cellular structures leading to lipid peroxidation and DNA oxidative damage, which are classical markers for cellular oxidative stress (18). By measuring lipid peroxidation, we found increased MDA levels in both SS and SH cells with respect to controls (+195 and +255%, respectively; *p* ≤ 0.001), which decreased upon incubation with silybin (−49 and −52% with respect to SS and SH cells, respectively; *p* ≤ 0.001) (Figure 7A). When extracellular levels of 8-OHdG were measured as a marker for oxidative DNA damage, a slight but no significant increase was observed in both SH and SS cells, and silybin reduced 8-OHdG levels in both conditions (−49% in SS and −50% in SH cells with respect to their counterparts; *p* ≤ 0.05) (Figure 7B).

The ability of silybin in protecting cells from fat-induced oxidative stress was confirmed by measuring the activity of the antioxidant enzyme catalase and expression of the transcription factor NF-κB, which is activated by oxidative stress. Catalase activity increased in both SS and SH cells with respect to control (+145% and +140%, respectively; *p* ≤ 0.01) and decreased upon incubation with silybin (−38% and −65% with respect to SS and SH cells, respectively; *p* ≤ 0.01 and *p* ≤ 0.001) (Figure 7C). NF-κB activation did not change significantly in SS and SH cells with respect to controls, but a significant reduction was observed upon exposure to silybin (−29% and −31% with respect to SS and SH cells, respectively; *p* ≤ 0.05) (Figure 7D).

## Discussion

By using a novel cellular model mimicking initiation and progression of NAFLD *in vivo*, we demonstrated that silybin provides important protective and anti-steatotic effects involving crucial pathways, especially at mitochondrial level. Our model paves the way to simultaneous assessment of morphological and functional characteristics of hepatocytes exposed to the steatogenic and pro-inflammatory hits, thus resulting an optimal and reliable system to test the beneficial and direct effects of silybin, a bioactive compound known for its vague “hepatoprotective” features.

Chronic caloric overload initiates an inflammatory response originating from the adipose tissue with production of cytokines, such as TNFα, that impairs lipid metabolism in remote tissues such as the liver thus promoting the progression of SS to SH (49). Therefore, in our model, we induced a condition mimicking NAFLD progression by exposing SS cell to TNFα for 24 h. NAFLD progression was ascertained by assessing: (i) cell viability, which was not altered in SS cells but was significantly reduced in SH cells; (ii) IkBip expression, a classical liver damage marker, which increased in SS cells and further increased in SH cells; and (iii) caspase activity, a marker of apoptosis, which was stimulated in SS cells and further stimulated in SH cells.

A first result of our study is that TG accumulation did not increase when our SS cells progressed to SH cells, thus suggesting that this experimental system might mimic the so-called burned-out NASH, a NASH sub-type in, which hepatic TG accumulation does not increase with the progression of disease (50). Interestingly, the progression of SS cells to SH cells was associated to a slightly reduction in LD size and these changes of LD morphometry were paralleled by alterations of ADRP, the main LD-associated protein, which promotes FA uptake and LD enlargement. In fact, ADRP expression increased in SS cells, but it was reduced in SH hepatocytes. Therefore, we can suggest that the worsening of hepatocyte condition in our *in vitro* model of NAFLD progression may depend on a fragmentation of LDs rather than on an increased fat content *per se*. Increased LD surface may facilitate the traffic of FAs across LDs and the intracellular environment. In line with this result, ATGL expression showed a very large increase in SH cells; this indicates a high rate of lipid mobilization in SH cells with harmful potentials. According to expectations, we observed a stimulation of TG secretion in SS cells, a likely attempt to protect cells from excess TG accumulation, while TG secretion was reduced in SH cells, and this impair in lipid release might worsen the damage in lipid homeostasis.

To limit excess fat accumulation, in both SS and SH cells, the systems involved in the mitochondrial oxidation of FAs were stimulated (CPT-1 expression), whereas microsomal (CYP2E1 expression) and peroxisomial (AOX activity) oxidation of FAs were stimulated only in SS cells. Over-active FA oxidation increases production of ROS with consequent oxidative stress, one of the key mechanisms responsible for NAFLD progression (17, 18). Indeed, in both SS and SH cells, we found a condition of oxidative stress as indicated by the increase in (i) the cellular level of MDA, marker of lipid peroxidation; (ii) the extracellular level of 8-OHdG, marker of oxidative damage, which is increased in patients with NASH with respect to SS and it was related to grade of inflammation; and (iii) the specific activity of catalase, the main antioxidant enzyme.

Impaired mitochondrial structure and function are reported to be a crucial event during progression of NAFLD. Ultrastructural analysis by EM showed that mitochondrial size was not significantly altered in both SS and SH cells compared to control cells. However, the mitochondrial cristae were largely disorganized and reduced in number in SH cells, and analysis of TMRE-stained mitochondria showed a reduced inner membrane polarization in SH cells, a further sign of damage (51). We observed a loss of mitochondria during progression from SS to SH, which was indicated by the marked reduction in the mtDNA copy number in SH cells. Also, the expression of APE1 and POLG, two main genes for mtDNA repair, was stimulated in SH cells. All these facts clearly indicate that mitochondria structure and number are largely damaged in our *in vitro* model of NAFLD progression.

Mitochondrial respiration parameters were assessed by using the Seahorse XF Analyzer. We did not observe significant differences in basal respiration in both SS and SH cells with respect to control, but the proton leak was significantly reduced in both SS and SH cells with respect to controls. This observation could indicate a decrease in mitochondrial uncoupling, possibly resulting from mitochondrial membrane alterations, which made it more impermeable to protons. In contrast, maximal respiration and ATP production did not change in SS cells, but they increased in SH cells, which is an expected finding since mitochondrial function would be expected to be damaged in NASH (52). Finally, in response to protonophore addiction (FCCP), SH cells showed increased respiration, indirectly suggesting higher activity of the respiratory chain. We wish to emphasize that progression from increased to deteriorated mitochondrial function has been also observed in the context of fatty liver disease (53). Our present biological model may recapitulate the up-regulation of mitochondrial function observed in the initial stages of the disease. This probably indicates that SH cells were more damaged than SS cells. Taken together, our data parallel previous studies conducted in patients with simple liver steatosis and biopsy-proven SH undergoing functional breath tests with stable isotope to assess mitochondrial function *in vivo* and showing that indeed SH is the worst functional *scenario* (54).

The nutraceutic silybin has shown preliminary encouraging results for NAFLD either in clinical and animal studies (55, 56). The present study aimed to verify and better understand the molecular mechanisms sustaining the beneficial action of silybin on the hepatocyte. A key finding is that silybin exerts hepatoprotective effects on both SS and SH cells, at different levels depending on the NAFLD grade. First of all, silybin was able to reduce the apoptosis observed in SS and SH cells, although it did not improve the viability compromised in SH cells. More specifically, in our cellular models of NAFLD progression we observed a significant anti-steatotic action of silybin. In particular, silybin treatment led to (i) marked reduction of excess TGs accumulated in both SS and SH cells and (ii) down-regulation of IkBip expression that resulted increased passing from control to SS till to SH cells. The lipid-lowering action of silybin in SS cells was associated to a decrease in LD size but not in LD number.

The lipid-lowering effect of silybin was sustained by a transcripitonal modulation of PPARs, the master regulators of lipogenic and lipolytic pathways (48). In both SS and SH cells, silybin reduced expression of PPARγ, the lipogenic isoform promoting esterification of FAs. PPARγ expression typically increases in NAFLD; indeed, it was up-regulated in both SS and SH cells. Regarding PPARα, an activator of mitochondrial and peroxisomal β-oxidation of FAs, it was only slightly up-regulated in SS and SH cells and silybin played different effects depending on the extension of cell damage. In SS cells, silybin increased PPARα expression, whereas in SH cells, it reduced it. Further studies need to clarify if such results imply distinct effects of silybin at different levels of steatogenic damage and play a role also *in vivo*.

The lipid-lowering effect of silybin was not supported by increased TG secretion, but by stimulation of FA oxidation. Expression of both mitochondrial CPT-1 and microsomal CYP2E1 was significantly up-regulated by silybin in both SS and SH cells. By contrast, the peroxisomial oxidation played by AOX was stimulated by silybin only in SH cells. The action of silybin appeared to be mainly dependent on its effects at the mitochondrial level, with different mechanisms depending on the NAFLD grade. In fact, at mitochondrial level, although silybin did not rescue the loss of mitochondria observed in SH cells, in both SS and SH cells, it led to a significant increase in mitochondria size (both major and minor axes), improved the organization of mitochondrial cristae, which became more evident and parallel to each other’s, and increased the polarization of the inner membrane of mitochondria as indicated by the increased and better defined TMRE signal. Moreover, silybin increased expression of the DNA repair enzymes APE1 and POLG and reduced both basal and maximal respiration only in SH cells, where their rates were increased, and this may stem from different factors including modulation of mitochondrial substrate import or decreased ATP production. Interestingly, silybin stimulated ATP production in SS cells but decreased it in SH cells, where the ATP production rate was already higher, and these changes paralleled those observed for PPARα expression. In contrast, silybin did not counteract the reduction of proton leak observed in both SS and SH cells. Although more work needs to be performed, silybin appears to act as an inhibitor of mitochondrial function under diseased conditions (especially in SH cells), which may have beneficial effects as observed previously with other agents (57). The apparent increased oxidative metabolism in SH cells may be justified by increasing anabolic pathways, as previously described (58).

Oxidative stress was observed in both SS and SH cells. Interestingly, the increased ROS generation following intracellular FA accumulation was almost completed blunted by co-treating the cells with silybin. In fact silybin was able to counteract the FA-dependent increase in (i) the lipid peroxidation measured as production of MDA; (ii) the oxidative DNA damage measured as extracellular levels of 8-OHdG; and (iii) the catalase activity. Excess ROS as well as pro-inflammatory cytokines can activate inflammatory signaling such as that sustained by the transcription factor NF-κB, which is widely implicated in the response to oxidative stress (20). The ability of silybin in protecting cells from fat-induced oxidative stress was confirmed by analyzing NF-κB activation, which was reduced upon exposure to silybin.

In conclusion, the present study provides new insights about the protective effects of silybin administered directly to hepatocytes mimicking *in vitro* the NAFLD progression. Further studies are on the way to translate such promising results into long-term beneficial effects, in the hope that onset, progression and worsening of NAFLD/NASH will be prevented/delayed in human being by using more natural nutraceutic approaches.

## Author Contributions

All authors contributed to this work significantly. GV performed cultures and treatments of cells, TG quantification, spectrophotometric experiments, mitochondrial respiration measurements, and fluorescence imaging of mitochondria and participated in writing the manuscript. EG carried out quantitative RT-PCR and optical microscopy experiments. FC performed immunoassay for 8-OHdG and quantification of mtDNA copy number. FB carried out lipid peroxidation and western blot analyses. PO supervised experiments of fluorescence imaging of mitochondria and oxygen consumption by Seahorse and participated in writing the manuscript. VS carried out oxygen consumption measurements by Seahorse and participated in writing the manuscript. KC carried out electron microscopy analysis. AL participated to 8-OHdG immunoassay and mtDNA analyses. AV participated in cell cultures and treatments. PP participated in conceiving and designing the study, supplied silybin and critical revised the manuscript. LV conceived and designed the study, analyzed and elaborated data, and wrote the manuscript. All authors have read, approved, and agreed to submit the manuscript to Frontiers in Phisiology; no part of the work has been published before.

## Conflict of Interest Statement

The authors declare that the research was conducted in the absence of any commercial or financial relationships that could be construed as a potential conflict of interest.

## Footnotes

^1^http://rsb.info.nih.gov/ij.

^2^http://www.ncbi.nlm.nih.gov/Genbank/GenbankSearch.html.
